# Long COVID among healthcare workers: a narrative review of definitions, prevalence, symptoms, risk factors and impacts

**DOI:** 10.1093/bmb/ldae008

**Published:** 2024-08-25

**Authors:** Brendan Dempsey, Ira Madan, Sharon A M Stevelink, Danielle Lamb

**Affiliations:** Department of Primary Care and Population Health, University College London, 1-19 Torrington Place, London, WC1E 7HB, UK; Guy’s and St Thomas NHS Foundation Trust, St Thomas' Education Centre, 75-79 York Road, London, SE1 7NJ, UK; Department of Psychological Medicine, Institute of Psychiatry, Psychology & Neuroscience, 16 De Crespigny Park, King’s College London, London, SE5 8AF, UK; Department of Primary Care and Population Health, University College London, 1-19 Torrington Place, London, WC1E 7HB, UK

**Keywords:** long COVID, post-COVID-19 syndrome, post-COVID-19 condition, ongoing symptomatic COVID, healthcare workers, healthcare staff

## Abstract

**Introduction:**

Long COVID (LC) occurs when people experience symptoms for weeks, months or even years after a COVID-19 infection. This review looks at research exploring the LC definitions, prevalence, symptoms, risk factors, and associated impacts in research on healthcare workers (HCWs).

**Data sources:**

We systematically searched five electronic databases (CINAHL, EMBASE, Medline, PsycInfo and PubMed) and compiled a narrative literature review based on 56 relevant studies.

**Areas of agreement:**

LC is prevalent among HCWs who become infected by COVID-19. Many of the most frequent symptoms associated with LC in the general population are also reported among HCWs. Some risk factors for LC are also similar to those in the general population, such as female sex, older age, and having a pre-existing respiratory illness.

**Areas of controversy:**

The mechanism(s) responsible for LC remains unknown. A variety of terms, timeframes and symptoms are used to define LC, creating difficulties in comparing results across studies. Much of the research is cross-sectional and fails to explore the impacts that prolonged symptoms have on HCWs’ personal and professional lives.

**Growing points:**

The need to support HCWs with LC is clear. Identifying the mechanism(s) responsible for LC is a key priority, as this will inform treatments.

**Areas for developing research:**

Future research should move towards a standard definition for LC. Greater attention should be paid to longitudinal and qualitative studies, which could give insights into prognosis, lived experience and work participation. Finally, studies evaluating treatments suitable for people with LC are timely.

## Introduction

Long COVID (LC) occurs when COVID-19 symptoms persist or develop after the acute phase of infection.^1–3^ Under the LC umbrella, there are a variety of definitions that differ primarily by the terms used to describe the condition and the length of time between symptom onset and persistence required to be considered LC.^4^ For example, the World Health Organization (WHO) defines post-COVID-19 condition as ‘the continuation or development of new symptoms three months after the initial SARS-CoV-2 infection, with these symptoms lasting for at least two months with no other explanation’.^3^ Meanwhile, the National Institute for Health and Care Excellence (NICE) differentiates between two kinds of LC: ongoing symptomatic COVID-19 (symptoms between 4–12 weeks after onset of COVID-19 infection) and post-COVID-19 syndrome (symptoms 12 or more weeks after onset of infection).^1^ The variety of available definitions creates challenges in comparing results across studies.^4^

LC was poorly understood throughout the early years of the pandemic,^5–7^ and more recent reviews remain unable to attribute a pathogenesis or prognosis for the condition, though several mechanisms have been theorized.^8–11^ The symptoms associated with LC have varied greatly, with one review suggesting that there may be as many as 60 symptoms.^5^ Among the most common of these are fatigue, shortness of breath, loss or change to sense of taste and/or smell, cognitive impairment, and issues with sleeping.^5^^,^^6^ This variety of symptoms creates difficulties in comparing results across studies, as different studies measure LC using different symptoms.^4^ Additionally, many of these symptoms may be associated with other conditions or may simply be common regardless of previous COVID-19 infection, likely making it difficult to ascertain which symptoms are directly caused by LC.

With regard to the prevalence of LC, conservative estimates suggest that approximately 10% of all people who contract COVID-19 will experience prolonged symptoms for 12 or more weeks following the onset of infection.^8^^,^^12^ Studies investigating the prevalence of LC have varied greatly, with a meta-analysis finding that studies estimating prevalence based on healthcare records predict significantly lower prevalence (13.6%; 95%CI 1.2%–68%) compared with studies using self-reported symptoms and their duration (43.9%; 95%CI 8.2%–87.2%).^13^ There may be several reasons for this difference, including an under-reporting of LC symptoms to healthcare professionals, difficulty accessing LC-healthcare services, lack of knowledge about LC, or differences in how LC may be diagnosed. Alternatively, people with LC symptoms simply may not seek diagnosis, believing that their symptoms are mild or that nothing can be done to help them. While the mechanisms responsible for LC are unknown, a meta-analysis of 41 studies identified several risk factors for prolonged symptoms for 12 or more weeks, including female sex, older age, having a pre-existing respiratory illness, being unvaccinated against COVID-19, being hospitalized during the acute COVID-19 infection, and a history of common mental disorders prior to COVID-19 infection.^14^

### LC among healthcare workers

Healthcare workers (HCWs) have been at an increased risk of COVID-19 infection throughout the pandemic.^15^ In the UK, research using data from the UK Office for National Statistics (ONS) COVID-19 Infection Survey found that HCWs were at significantly higher risk of contracting COVID-19 compared with non-essential workers during the first year of the pandemic, though this effect reversed by June 2021.^16^ Another study using ONS data found that HCWs were among the occupational groups to report significantly higher prevalence of symptoms for four or more weeks.^17^ Research using ONS mortality data found that HCWs were significantly more likely to die as a result of COVID-19 in the first year of the pandemic compared to other industries, with this effect diminishing thereafter^18–20^. The fall in the prevalence of COVID-19 and COVID-19-related mortality among HCWs may be related with high levels of vaccination during 2021^16^^,^^18^^,^^20^, as well as the emergence of mutations of COVID-19 less likely to cause serious infection^18^. Given the increased risk of COVID-19 infection among HCWs, particularly in the first year of the pandemic, they are presumably at high risk of LC. We also acknowledge that this is likely not unique to HCWs, as several other occupational groups, e.g. educators, bus and coach drivers, and police and protective services staff, have also been found to be at an increased risk of COVID-19 infection in the UK compared with the general population.^15^^,^^16^

### Aim

The main aim of this study was to review the literature surrounding LC in HCWs and describe the definitions used, prevalence, symptoms, risk factors and impact on HCWs.

## Methods

In line with our aim, a narrative review was used to broadly summarize what has been previously published in this area, identifying knowledge gaps, common findings, and expected areas of heterogeneity. We did not conduct a systematic review, which includes a meta-analysis, on the studies included in the review due to the expected heterogeneity of measures and LC-related outcomes, but acknowledge an ongoing meta-analysis in this area.^21^

### The search strategy

The search strategy was designed using recommendations from the Joanna Briggs Institute.^22^ The PCC mnemonic (Population, Context, Concept) was used to build the search string (see Appendix 1 in the Supplementary Materials). A three-step process was used to search for articles.^22^ The first step was to build a basic search string, use it in a database (OVID), and then expand on it using keywords and MeSH terms. The second step was to use the updated string in five electronic databases: CINAHL (via EBSCO), EMBASE (via OVID), Medline (via OVID), PsycInfo (via OVID) and PubMed (via NCBI). The final step was to search again in each of the electronic databases to identify newly published sources.

### Screening eligible studies

The lead author designed the eligibility criteria and then met with the co-authors to discuss. The inclusion and exclusion criteria were then finalized and are presented in Appendix 2 in the Supplementary Materials. In summary, English language, original research articles were included if they examined LC in a sample of HCWs. No restrictions were set regarding study methodology or design, as long as the article discussed the prevalence, nature, risk factors, lived experience and/or impacts associated with LC among a sample of HCWs. Pre-print articles were included if they reported the results of original research, but sources such as editorials, commentaries, newspaper articles, and case reports were excluded. Papers were also excluded if they reported on original research conducted with a sample, which included HCWs but did not clearly distinguish these individuals from non-HCWs.

The citations retrieved by the database searches were uploaded to Rayyan. After removing duplicate records, the lead author conducted a title, abstract and then a full-text review on the citations, in line with the eligibility criteria. The final author conducted an independent title review on 10% of the citations. There was a 99% agreement between both reviews, and we reached 100% agreement after meeting and discussing the conflicts. The full-text review was conducted in Zotero.

### Charting, data extraction and synthesis

Charting was conducted alongside the full-text review. To aid this process, the lead author developed a spreadsheet, which included basic information such as study reference information, context, and design, and a summary of the results from each study, which were sorted into categories based on the review’s aims (e.g. how did the studies define LC and what was the prevalence of developing LC in the sample; see Appendix 3 in the Supplementary Materials). These categories were devised and refined by the research team following a pilot exercise with ten articles. To combine the results, the charted data within each category were sorted theoretically to curate sub-categories. Narrative summaries explaining the convergences and divergences within these data were then created for each category. Where numeric data were available, e.g. the prevalence of LC or of specific symptoms, we have conducted descriptive statistics to give an overview. For data on the prevalence of LC, we present the median value, along with the inter-quartile range across all studies. For symptoms, we present the median value, along with the highest and lowest observed values across the studies. All analyses were conducted using Microsoft Excel. Where results of formal analytical tests were presented in the study, e.g. *P* values for tests examining risk factors for LC, we highlight which values were significant, along with the direction of effect.

## Results

**Fig. 1:**
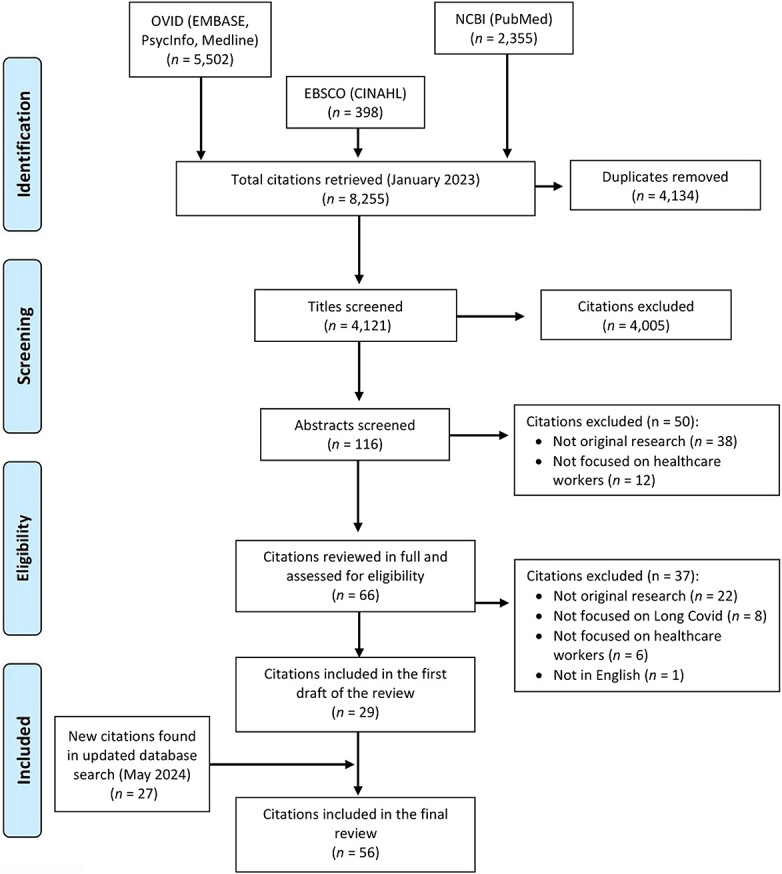
Flow chart showing the study selection process for the literature review, adapted from the free chart designed by PRISMA.

The first database search, conducted in January 2023, identified a total of 4121 unique citations. Following the title screening, 4005 were excluded due to irrelevance. The abstracts of the remaining 116 were reviewed, with 66 of these being inspected in full. A total of 29 articles were included in the first draft of the review.^23–51^ The second database search was conducted in May 2024, and an additional 27 studies were included in the review.^52–78^ See Figure 1 for an overview of the screening process.

### Study characteristics

Of the 56 included articles, 52 were quantitative, two were qualitative, and two were mixed methods. The studies reported on a total sample of 60 810 HCWs working in 45 named countries. Also included were three unnamed countries (one study collected data in 21 Latin American countries, though only named the 18 countries from which they received 10 or more responses^75^). Approximately, half of the studies come from Europe (*n* = 25), with the rest coming from Asia (*n* = 18), Africa (*n* = 6), South America (*n* = 6) and North America (*n* = 1). For the quantitative studies, the sample sizes ranged from the smallest of 30^38^ to the largest of 25 516^58^ (median 243, IQR 124.5, 547.5). The two qualitative studies sampled 13^44^ and 43^30^ HCWs, respectively, while the mixed methods studies included 120^26^ and 471^77^ HCWs. The majority of the studies (*n* = 44) employed a cross-sectional design,^23–27^^,^^30–32^^,^^36–44^^,^^47–58^^,^^60–62^^,^^64–67^^,^^70^^,^^72–78^ while the remaining 12 were longitudinal.^28^^,^^29^^,^^33–35^^,^^45^^,^^46^^,^^59^^,^^63^^,^^68^^,^^69^^,^^71^

### Defining LC

All the studies measured LC by the presence of persistent or new COVID-19 symptoms after the acute phase of the illness. None of the studies specified that HCWs must have experienced multiple symptoms or that specific symptoms, e.g. fatigue, must be present to be diagnosed with LC.

A variety of guidelines and terms were used to define LC. Eleven studies cited the NICE guideline,^34^^,^^44^^,^^46^^,^^48^^,^^56–58^^,^^65^^,^^72^^,^^75^^,^^77^ which differentiates between two different types of LC: ongoing symptomatic COVID-19 (OSC), which is signs and symptoms of COVID-19 from four weeks up to 12 weeks after initial infection, and post-COVID-19 syndrome (PCS), which includes signs and symptoms of COVID-19 12 or more weeks from initial infection.^1^ Another nine studies cited the WHO guidelines on defining LC,^33^^,^^37^^,^^55^^,^^61^^,^^62^^,^^69^^,^^70^^,^^75^^,^^78^ which defines post-COVID-19 condition as ‘the continuation or development of new symptoms three months after the initial SARS-CoV-2 infection, with these symptoms lasting for at least two months with no other explanation’.^3^ Three studies cited Greenhalgh et al.,^39^^,^^40^^,^^49^ who define post-acute COVID-19 as ‘extending beyond three weeks from the onset of first symptoms’ and chronic COVID-19 as ‘extending beyond 12 weeks’.^79^ Guidelines that were cited only once included those by the Centre for Disease Control and Prevention,^57^ the National Centre for Immunization and Respiratory Diseases,^68^ the Mayo Clinic,^59^ Tantipasawasin et al.,^76^ the Indonesian Society of Respirology,^74^ and the German Society of Pneumology.^53^ The remaining 29 studies did not cite a specific guideline when defining LC.^23–32^^,^^35^^,^^36^^,^^38^^,^^41–43^^,^^45^^,^^47^^,^^50–52^^,^^54^^,^^60^^,^^63^^,^^64^^,^^66^^,^^67^^,^^71^^,^^73^

As few studies cited official guidelines, there were several differences in how the studies operationalized LC. Notably, many differed by the length of time between the onset of COVID-19 symptoms and data collection. Thirty-eight studies specified a single length of time that COVID-19 symptoms must be experienced to be considered LC. Among these, 15 defined LC as the presence of COVID-19 symptoms 12 weeks/three months after the initial infection.^23^^,^^26^^,^^33–35^^,^^37^^,^^38^^,^^42^^,^^48^^,^^49^^,^^55^^,^^61^^,^^69^^,^^70^^,^^78^ Twelve studies defined LC as symptoms that extended for more than four weeks/one month after initial infection.^25^^,^^43^^,^^51^^,^^54^^,^^56^^,^^58^^,^^64^^,^^66^^,^^68^^,^^72^^,^^73^^,^^76^ Five studies measured symptoms six months or more after initial infection with COVID-19,^29^^,^^45–47^^,^^67^ one measured symptoms two years after the initial COVID-19 infection,^71^ and one study defined LC as symptoms 3 or more weeks after the acute COVID-19 infection.^30^ The remaining four studies did not explicitly specify a necessary time between symptom onset and continuation when defining LC, though noted that they collected data three months after the initial infection.^36^^,^^44^^,^^50^^,^^52^

Fourteen studies made a distinction between different stages of LC. Five studies differentiated between a preliminary stage (i.e. symptoms between 4 and 12 weeks after initial infection) and a chronic stage of LC (i.e. symptoms 12 weeks or more after initial infection).^28^^,^^40^^,^^62^^,^^74^^,^^75^ A sixth chose a similar categorization, but defined the preliminary stage of LC as persistent symptoms between 3 and 12 weeks after initial infection.^39^ The remaining studies differentiated between persistent symptoms after initial infection at one, three and six months,^27^^,^^31^^,^^63^^,^^65^ one, three, six and twelve months,^77^ one, three, six, nine and twelve months,^59^ one and two months,^57^ or six and twelve months.^60^

The remaining four studies did not define LC and did not give a clear indication of the time between the onset of acute COVID-19 symptoms and data collection.^24^^,^^32^^,^^41^^,^^53^

### Prevalence of LC and its symptoms

Forty-two studies estimated the prevalence of LC in their samples. Of the 14 other studies, 8 only collected data among HCWs experiencing LC, therefore having 100% prevalence in their sample,^24^^,^^30^^,^^32^^,^^33^^,^^44^^,^^56^^,^^75^^,^^77^ and the remaining 6 did not clearly report the prevalence of LC in the sample.^28^^,^^40^^,^^45^^,^^47^^,^^51^^,^^53^ We followed the NICE guidelines^1^ to explore the prevalence of OSC (symptoms for 4+ weeks) and PCS (symptoms for 12+ weeks) across the included studies.

**Fig. 2:**
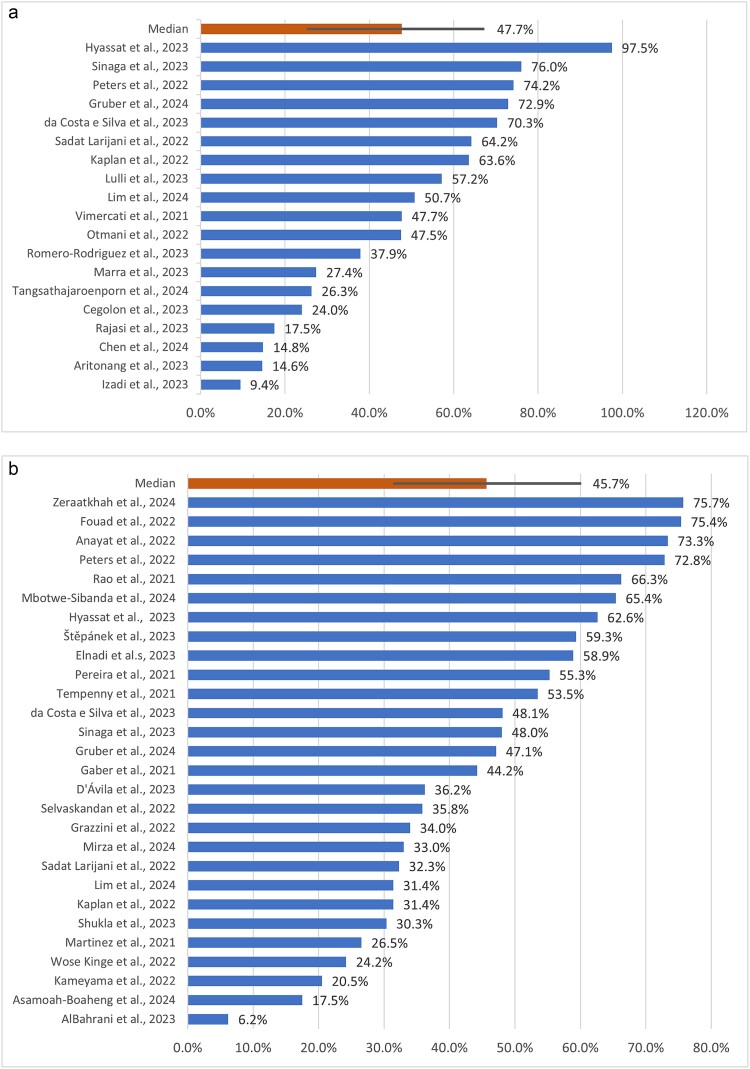
**(a)** Prevalence of ongoing symptomatic Covid-19 (symptoms for four or more weeks after the onset of Covid-19 infection) among the studies included in the review. **(b)** Prevalence of post-Covid-19 syndrome (symptoms for 12 or more weeks after the onset of Covid-19 infection) among the studies included in the review. Bars on the median values show the inter-quartile range.

OSC was reported in 19 studies.^25^^,^^27^^,^^34^^,^^39^^,^^43^^,^^54^^,^^57–59^^,^^62–66^^,^^68^^,^^72–74^^,^^76^ The median prevalence of OSC was 47.7% (IQR 25.2–67.3%), and individual studies ranged from a low of 9.4% (42 of 445 HCWs)^64^ to a high of 97.5% (136 of 140 HCWs).^63^ PCS was reported in 28 studies.^23^^,^^26^^,^^27^^,^^29^^,^^31^^,^^34–39^^,^^41^^,^^42^^,^^46^^,^^48–50^^,^^52^^,^^55^^,^^59^^,^^61–63^^,^^65^^,^^69^^,^^70^^,^^74^^,^^78^ The median prevalence of reporting PCS was 45.7% (IQR 31.4–60.2%), with studies that ranged from a low of 6.2% (15 of 243 HCWs)^52^ to a high of 75.7% (265 of 350 HCWs).^78^ See Figure 2A and B for an overview of the prevalence of OSC and PCS across the studies.

Forty-one studies reported on a total of 76 LC symptoms present in a sample of 12 260 HCWs. Of the studies that did not report symptoms, two were qualitative and did not describe the proportion of participants reporting symptoms,^30^^,^^44^ while the remaining 13 either did not report symptoms or did not clearly define what proportion of the sample was diagnosed with LC.^25^^,^^40^^,^^45^^,^^47^^,^^51^^,^^56–59^^,^^62^^,^^64^^,^^70^^,^^76^ Of the 41 studies, 26 reported 10 or more symptoms, while 10 studies reported 20 or more symptoms. We have sorted the symptoms by organ system involved based on previous studies in this area.^6^ The supporting references for each symptom can be found in Table 1.

**Table 1:** The Long Covid symptoms and their prevalence reported by healthcare workers in the studies included in the literature review

**Organ system and symptoms**	**Median** ^*^	**Low** ^**^	**High** ^**^	**Number of studies and references**
**General symptoms (14)**				
Quality of life	65.8	31.5^28^	100.0^29^	2^28^^,^^29^
Fatigue	53.7	2.4^60^	100.0^61^	40^23^^,^^24^^,^^26–29^^,^^31–39^^,^^41–43^^,^^46^^,^^48–50^^,^^52–55^^,^^60^^,^ ^61^^,^^63^^,^^65^^,^^66^^,^^68^^,^^69^^,^^71–75^^,^^77^^,^^78^
Climbing stairs and muscular strain	52.6			1^53^
Not feeling well	45.3			1^78^
Chills and shivering	43.4			1^73^
Lower strength	36.6			1^65^
Postexertional malaise	36.5	2.4^60^	70.0^38^	7^35^^,^^38^^,^^55^^,^^60^^,^^63^^,^^73^^,^^77^
Physical fitness	35.6			1^28^
General health	28.8			1^28^
Hypothermia	27.0			1^73^
Motivation	20.2	16.2^65^	24.1^53^	2^53^^,^^65^
Weight loss	17.1	6.7^52^	24.5^27^	4^27^^,^^38^^,^^52^^,^^65^
Fever	8.8	0.6^42^	55.9^73^	14^23^^,^^27^^,^^34^^,^^35^^,^^38^^,^^41^^,^^42^^,^^55^^,^^60^^,^^65^^,^^68^^,^^73^^,^^77^^,^^78^
Loss of sex drive	7.3	6.3^65^	8.3^78^	2^65^^,^^78^
**Respiratory symptoms (10)**				
Hoarseness	33.6			1^73^
Aphonia	31.1	27.9^78^	34.2^73^	2^73^^,^^78^
Pneumonia	26.3			1^73^
Dyspnea/shortness of breath	24.6	4.5^55^	87.0^33^	34^23^^,^^24^^,^^27–29^^,^^33–39^^,^^41^^,^^43^^,^^46^^,^^49^^,^^50^^,^^53^^,^^55^^,^^60^^,^^61^^,^ ^63^^,^^65^^,^^66^^,^^68^^,^^69^^,^^71–75^^,^^77^^,^^78^
Tightness in the chest	23.0	7.3^23^	38.8^73^	2^23^^,^^73^
Chest pain	17.3	2.7^49^	33.3^52^	20^27^^,^^32^^,^^35^^,^^37–39^^,^^42^^,^^43^^,^^49^^,^^52^^,^^55^^,^^61^^,^^63^^,^ ^65^^,^^71–73^^,^^75^^,^^77^^,^^78^
Cough	16.9	2.4^60^	63.3^38^	32^23^^,^^27^^,^^29^^,^^31^^,^^33–35^^,^^38^^,^^39^^,^^41–43^^,^^49^^,^^50^^,^^52–55^^,^ ^60^^,^^61^^,^^63^^,^^65^^,^^66^^,^^68^^,^^69^^,^^71–75^^,^^77^^,^^78^
Wheezing	13.3			1^52^
Phlegm	6.6	2.6^39^	28.9^73^	4^27^^,^^39^^,^^49^^,^^73^
Pulmonary fibrosis	1.8			1^27^
**Ear, nose and throat symptoms (10)**				
Anosmia/loss or change of smell	26.9	3.0^74^	57.9^73^	27^23^^,^^26^^,^^27^^,^^31^^,^^33–35^^,^^38^^,^^39^^,^^41–43^^,^^46^^,^^49^^,^^53^^,^ ^55^^,^^60^^,^^61^^,^^63^^,^^65^^,^^66^^,^^71^^,^^73–75^^,^^77^^,^^78^
Rhinitis	23.4	12.0^34^	34.9^73^	2^34^^,^^73^
Ageusia/loss or change of taste	18.0	2.7^49^	51.1^35^	23^23^^,^^26^^,^^27^^,^^33–35^^,^^38^^,^^39^^,^^41–43^^,^^49^^,^^52^^,^^53^^,^^60^^,^^61^^,^ ^63^^,^^65^^,^^66^^,^^71^^,^^73^^,^^77^^,^^78^
Tinnitus	14.3	1.0^23^	19.3^77^	5^23^^,^^43^^,^^65^^,^^75^^,^^77^
Dry mouth	12.1			1^78^
Unpleasant smell	11.7			1^78^
Congestion/runny nose	11.2	0.6^42^	45.1^68^	6^27^^,^^34^^,^^39^^,^^42^^,^^68^^,^^73^
Sore throat	10.4	5.4^49^	31.3^78^	12^23^^,^^27^^,^^34^^,^^38^^,^^41^^,^^49^^,^^55^^,^^68^^,^^71^^,^^73^^,^^77^^,^^78^
Earache	4.3	1.5^23^	11.5^77^	3^23^^,^^27^^,^^77^
Hearing loss	4.3			1^27^
**Neurological and cognitive symptoms (13)**				
Brain fog^***^	32.4	1.1^50^	70.7^48^	12^34^^,^^37^^,^^42^^,^^43^^,^^48^^,^^50^^,^^55^^,^^63^^,^^65^^,^^67^^,^^71^^,^^77^
Headache	29.8	1.0^74^	99.1^61^	30^23^^,^^27^^,^^33–35^^,^^38^^,^^39^^,^^41–43^^,^^49^^,^^50^^,^^52^^,^^53^^,^^55^^,^^60^^,^ ^61^^,^^63^^,^^65–69^^,^^71–75^^,^^77^^,^^78^
Memory loss/confusion	25.1	5.4^42^	59.2^75^	13^23^^,^^27^^,^^28^^,^^33^^,^^38^^,^^39^^,^^52^^,^^53^^,^^60^^,^^61^^,^^74^^,^^75^^,^^78^
Difficulty concentrating	21.6	2.4^60^	45.4^75^	15^23^^,^^27^^,^^35^^,^^39^^,^^46^^,^^49^^,^^52^^,^^53^^,^^60^^,^^61^^,^^65^^,^^66^^,^^74^^,^^75^^,^^78^
Difficulty sleeping/insomnia	20.0	2.9^72^	59.4^32^	29^23^^,^^27^^,^^29^^,^^32^^,^^33^^,^^35–39^^,^^42^^,^^43^^,^^48^^,^^49^^,^^52–55^^,^^61^^,^ ^63^^,^^65–67^^,^ ^71^^,^^72^^,^^74^^,^^75^^,^^77^^,^^78^
Vertigo	16.2			1^78^
Dizziness	16.0	1.1^42^	44.7^73^	14^27^^,^^35^^,^^42^^,^^43^^,^^49^^,^^55^^,^^61^^,^^63^^,^^66^^,^^67^^,^^72^^,^^73^^,^^75^^,^^77^
Balance and fine motor skills	13.0			1^53^
Tremors	10.3			1^61^
Vision impairment	6.8	1.7^42^	29.6^73^	6^38^^,^^42^^,^^50^^,^^65^^,^^67^^,^^73^
Nightmares	5.6	5.4^49^	5.8^65^	2^49^^,^^65^
Paraesthesia/numbness	5.6	0.6^42^	29.5^77^	8^23^^,^^27^^,^^42^^,^^55^^,^^63^^,^^67^^,^^75^^,^^77^
Itchy eyes	2.7	1.1^42^	13.9^68^	3^42^^,^^47^^,^^68^
**Mental health symptoms (7)**				
Anxiety	21.5	0.5^23^	47.0^33^	18 ^23^^,^^24^^,^^27^^,^^33^^,^^37^^,^^41^^,^^43^^,^^48^^,^^49^^,^^52^^,^^53^^,^^61^^,^^65^^,^^71^^,^^73^^,^^75^^,^^77^^,^^78^
Mental exhaustion	21.0			1^71^
Irritable	19.5			1^53^
Mood swings	17.8	10.8^53^	44.0^36^	6^26^^,^^27^^,^^36^^,^^53^^,^^61^^,^^63^
Stress	16.8			1^71^
Anorexia	16.7	9.0^27^	30.9^78^	3^27^^,^^38^^,^^78^
Depression	14.1	2.9^42^	47.0^33^	15^23^^,^^27^^,^^35^^,^^37^^,^^41^^,^^42^^,^^48^^,^^49^^,^^52^^,^^61^^,^^65^^,^^71^^,^^75^^,^^77^^,^^78^
**Musculoskeletal symptoms (6)**				
Muscle weakness	49.1			1^78^
Musculoskeletal pain	29.6	23.6^66^	35.6^65^	2^65^^,^^66^
Myalgia/muscle aches and pains	28.5	3.0^74^	67.2^61^	24 ^23^^,^^27^^,^^34^^,^^35^^,^^37^^,^^39^^,^^42^^,^^43^^,^^50^^,^^52^^,^^53^^,^^55^^,^^60^^,^^61^^,^^63^^,^ ^68^^,^^69^^,^^71–75^^,^^77^^,^^78^
Arthralgia/joint pain	28.5	2.0^74^	72.0^33^	19 ^23^^,^^33–35^^,^^37^^,^^39^^,^^41–43^^,^^49^^,^^53^^,^^61^^,^^63^^,^^68^^,^^71^^,^^73^^,^^74^^,^^77^^,^^78^
Neck pain	7.3	4.2^65^	10.5^71^	2^65^^,^^71^
Back pain	5.5	2.6^39^	46.7^73^	4^39^^,^^47^^,^^60^^,^^73^
**Cardiovascular symptoms (5)**				
Palpitations	15.6	2.2^42^	48.2^77^	15^23^^,^^27^^,^^33^^,^^35^^,^^42^^,^^43^^,^^49^^,^^52^^,^^53^^,^^55^^,^^63^^,^^65^^,^^71^^,^^75^^,^^77^
Tachycardia	8.0	3.9^23^	17.4^78^	3^23^^,^^27^^,^^78^
Arrhythmia	6.1			1^27^
Sweating	4.3	0.6^42^	36.2^73^	3^27^^,^^42^^,^^73^
Hypertension	1.8			1^27^
**Dermatological symptoms (4)**				
Hair loss	24.0	2.4^60^	41.0^33^	10^27^^,^^33^^,^^35^^,^^38^^,^^42^^,^^52^^,^^60^^,^^69^^,^^71^^,^^72^
Eczema	15.8			1^73^
Itching	13.6	5.5^27^	21.7^73^	2^27^^,^^73^
Skin rash	3.4	2.2^42^	13.6^77^	7^23^^,^^42^^,^^49^^,^^65^^,^^71^^,^^77^^,^^78^
**Gastrointestinal symptoms (7)**				
Diarrhea	12.5	0.6^42^	43.4^73^	12^23^^,^^34^^,^^42^^,^^43^^,^^52^^,^^53^^,^^55^^,^^60^^,^^68^^,^^73^^,^^75^^,^^77^
Difficult swallowing	9.5			1^60^
Constipation	9.0	2.6^39^	13.3^52^	3^39^^,^^52^^,^^53^
Digestive problems	7.5	4.4^35^	10.5^71^	2^35^^,^^71^
Appetite change	7.2	4.4^23^	37.5^73^	8^23^^,^^35^^,^^49^^,^^53^^,^^71^^,^^73^^,^^75^^,^^77^
Nausea/vomiting	6.3	2.8^53^	30.3^73^	9^23^^,^^27^^,^^34^^,^^53^^,^^65^^,^^68^^,^^73^^,^^75^^,^^77^
Abdominal pain	5.7	1.1^42^	34.2^73^	10^23^^,^^27^^,^^34^^,^^38^^,^^42^^,^^43^^,^^49^^,^^55^^,^^73^^,^^77^^,^^78^

^*^Represents the median prevalence of each symptom across all studies that reported it. Where a symptom was reported by only one study, the prevalence of the symptom in that study is given. Median values have not been adjusted for the sample size in each study.

^**^The lowest and highest reported prevalence of each symptom, along with a reference to the study.

^***^Brain fog was defined as a combination of difficulty concentrating, memory loss, and confusion.

None of the symptoms were measured in every study, though fatigue was included in 40 (97.6%). It ranged from a low of 2.4^60^ to a high of 100.0,^61^ and with a median of 53.7%. The next most reported symptoms were dyspnea/shortness of breath (34 studies), cough (32 studies), headache (30 studies), difficulty sleeping/insomnia (29 studies), anosmia/loss or change of smell (27 studies), myalgia/muscles aches and pains (24 studies), and ageusia/loss or change of taste (23 studies). The remaining 68 symptoms were reported in fewer than 50% of the studies.

### Risk factors for LC

Thirty-two studies explored 35 potential risk factors for LC. Twenty-two tested for risk factors using regression modelling,^23^^,^^26^^,^^27^^,^^32^^,^^34^^,^^35^^,^^37^^,^^39^^,^^40^^,^^42^^,^^51^^,^^52^^,^^58^^,^^61–64^^,^^66^^,^^68^^,^^73^^,^^78^ while three used chi-square tests,^65^^,^^67^^,^^72^ two used Mann Whitney U tests,^25^^,^^50^ and then one each used a log-rank test,^33^ ANOVA,^69^ Fishers Exact test,^46^ Cox proportional hazards,^45^ or did not describe what test they used.^43^ Where studies included both univariable and multivariable regression analyses, we present the results of the multivariable models. We have sorted the risk factors into four categories: demographic risk factors, health risk factors, COVID-19 specific risk factors and occupational risk factors (see Table 2). Sex, age and weight were the only risk factors explored in more than half of the studies, with sex included in 26 studies, age in 23 and weight in 17. Of all risk factors explored in multiple studies, being infected by the Delta variant of the SARS-CoV-2 virus was the only factor to be unanimously statistically significantly associated with greater odds of reporting LC.^25^^,^^35^^,^^43^ Where a statistically significant association was observed in multiple studies, no study directly conflicted with another, e.g. eight studies found that females were at higher risk of developing LC, while none found that males were at higher risk.

**Table 2:** Risk factors for LC among healthcare workers explored by the studies in the literature review

**Risk factors & number of studies**		**Notes of significance**
**Demographic risk factors (5)**		
Sex	26	Eight studies found that female HCWs were at higher risk of developing LC,^23^^,^^34^^,^^42^^,^^50^^,^^63^^,^^65^^,^^68^^,^^70^ while 18 found no significant association^25–27^^,^^32^^,^^33^^,^^35^^,^^37^^,^^39^^,^^43^^,^^45^^,^^46^^,^^51^^,^^52^^,^^61^^,^^62^^,^^64^^,^^66^^,^^69^
Age	23	Ten studies found that older HCWs were at higher risk of developing LC,^27^^,^^34^^,^^35^^,^^37^^,^^40^^,^^42^^,^^50^^,^^61^^,^^64^^,^^68^ while 13 found no significant association^23^^,^^25^^,^^26^^,^^32^^,^^33^^,^^39^^,^^43^^,^^45^^,^^51^^,^^62^^,^^63^^,^^65^^,^^69^
Ethnicity	5	One study found that non-white HCWs were at higher risk of developing LC,^26^ while four found no significant association^37^^,^^46^^,^^51^^,^^65^
Nationality	1	One study found that Egyptian HCWs were at higher risk of developing LC compared to Cameroonian, Nigerian and Somali colleagues^61^
Marital status	1	One study found no significant association between marital status and developing LC^32^
**Health risk factors (21)**		
Weight	17	Three studies found that HCWs with higher BMI/who were overweight or obese were at higher risk of developing LC,^25^^,^^51^^,^^73^ while 14 found no significant association^23^^,^^27^^,^^32^^,^^34^^,^^35^^,^^39^^,^^42^^,^^45^^,^^63–66^^,^^68^^,^^70^
Pre-existing health condition (unspecified)	11	Three studies found that HCWs with any pre-existing health condition (unspecified) prior to contracting COVID-19 infection at higher risk of developing LC,^27^^,^^34^^,^^51^ while eight found no significant association^23^^,^^27^^,^^33^^,^^39^^,^^42^^,^^45^^,^^64^^,^^68^
Respiratory disease	8	Five studies found that HCWs with HCWs with respiratory disease prior to COVID-19 infection at higher risk of developing LC,^25^^,^^35^^,^^43^^,^^65^^,^^73^ while three found no significant association^52^^,^^63^^,^^70^
Hypertension	8	Eight studies found no significant association between hypertension and developing LC^25^^,^^35^^,^^45^^,^^52^^,^^63^^,^^65^^,^^70^^,^^73^
Diabetes	4	Four studies found no significant association between diabetes and developing LC^52^^,^^63^^,^^65^^,^^70^
Hypothyroid	1	One study found no significant association between hypothyroidism and developing LC^70^
HIV	1	One study found no significant association between HIV and developing LC^65^
Cancer	1	One study found no significant association between cancer and developing LC^65^
Cardiovascular disease	3	Three studies found no significant association between cardiovascular disease and developing LC^52^^,^^63^^,^^65^
Neurological disorder	1	One study found no significant association between neurological disorders and developing LC^63^
Allergy	1	One study found no significant association between allergies and developing LC^63^
Post-transplant	1	One study found that HCWs who had recently received an organ transplant were at higher risk of developing LC^52^
Depression	2	One study found that HCWs with a history of depression or state of exhaustion were at higher risk of developing LC,^35^ while one found no significant association^73^
Anxiety	1	One study found that HCWs with anxiety were at higher risk of developing LC^73^
Smoking	6	Six studies found no significant association between smoking and developing LC^25^^,^^35^^,^^45^^,^^63^^,^^65^^,^^70^
Alcohol intake	3	One study found that HCWs with higher alcohol intake were at higher risk of developing LC,^70^ while two found no significant association^37^^,^^51^
Recreational drug use	1	One study found no significant association between recreational drug use and developing LC^37^
Taking medication	1	One study found that HCWs taking medication were at higher risk of developing LC^51^
Sleep quality	1	One study found that HCWs with HCWs with worse sleep quality were at higher risk of developing LC^32^
Blood group	1	One study found that HCWs with B-type blood were at higher risk of developing LC^70^
Stress	1	One study found that HCWs under greater self-reported stress were at higher risk of developing LC^79^
**COVID-19 specific risk factors (7)**		
Symptoms during acute infection	5	Three studies found that HCWs with more severe symptoms during the acute COVID-19 infection were at higher risk of developing LC,^23^^,^^27^^,^^34^ while two found no significant association^42^^,^^61^
Vaccination	5	One study found that HCWs who were not vaccinated against COVID-19 were at higher risk of developing LC,^68^ while four found no significant association^23^^,^^45^^,^^51^^,^^70^^,^^72^
Treatment during acute infection	4	One study found that HCWs who received corticosteroids during acute infection at higher risk of developing LC,^78^ while three found no association between treatment during the acute infection and developing LC^23^^,^^32^^,^^34^
Number of acute COVID-19 infections	3	Two studies found that HCWs with more COVID-19 infections were at higher risk of developing LC,^65^^,^^68^ while one study found no significant association^70^
Number of symptoms	2	One study found that HCWs with more symptoms during the acute COVID-19 infection were at higher risk of developing LC,^78^ while one study found no significant association^42^
COVID variant	2	One study found that HCWs infected by Alpha/Delta or Wild variants at higher risk of developing LC,^51^ while another found that HCWs infected by Delta or Omicron variants were at higher risk^68^
N95 mask use	1	One study found that HCWs who did not use N95 masks in the workplace were at higher risk of developing LC^64^
**Occupational risk factors (2)**		
Job title	11	One study found that healthcare assistants at higher risk of developing LC,^50^ one study found that nurses and operating assistants were at higher risk,^67^ one study found that nurses were at higher risk,^78^ while eight found no significant associations^23^^,^^26^^,^^32^^,^^35^^,^^39^^,^^51^^,^^61^^,^^62^
Contact with COVID-19 patients	2	One study found that HCWs who have contact with COVID-19 patients were at higher risk of developing LC,^51^ while one found no significant assocation^62^

HCWs = healthcare workers; LC = Long COVID; significant association = *P* < 0.05 in the conducted statistical test; HIV = human immunodeficiency virus; BMI = body mass index; N95 respirator

### Potential impacts of LC

Seven of the 12 longitudinal studies reported impacts of LC.^28^^,^^29^^,^^33–35^^,^^63^^,^^71^ Of these, one found that HCWs with LC reported significantly lower physical and mental health compared to HCWs who were infected by COVID-19 but did not experience prolonged symptoms.^34^ Five studies found that HCWs with LC reported reduced general health,^28^ quality of life,^28^^,^^29^^,^^71^ and workability^33^^,^^34^ and increased functional impairment^71^ compared to a time before they became infected by COVID-19. Female HCWs in a Jordanian study reported negative changes to their menstrual cycle.^63^ As for work participation, a Swiss study found that 69 HCWs with PCS required a cumulative total of 1412 missed workdays (median 15, IQR 10–21)^35^ and a German study found that 107 HCWs (5.2% of the sample) had not returned to work after a COVID-19 infection.^34^

Cross-sectional research also identified some impacts related to LC. Compared to HCWs who were infected by COVID-19 but did not experience prolonged symptoms, six studies found increased functional impairments^47^^,^^61^^,^^67^ and burnout,^26^ and lower quality of life^53^ and workability^76^ among HCWs with LC. Of work participation, an Italian study found that 168 HCWs who became infected by COVID-19 (47.7% of the sample) required 35 or more days off work,^25^ a study which included HCWs from 21 Latin American countries found that 16% required a modification before returning to work,^75^ a study from the UK found that 18% of doctors with LC had not returned to work due to illness, while 40% of those who had returned to work required a phased return, with reduced hours or duties,^56^ and another UK-based study found that three HCWs (4.9% of those who had PCS) required a period of additional sick leave following their initial isolation period,.^36^ Furthermore, 69% of HCWs with PCS in the latter study felt that they were struggling to cope with their symptoms^36^.

Both qualitative studies, conducted in 2020 in the UK, provided more information on the HCWs’ lived experience of LC. One study included a range of HCWs,^30^ while the second sampled only doctors.^44^ In both studies, an uncertainty regarding persistent symptoms was emphasized, and HCWs described using their medical knowledge to make sense of their symptoms, with some fearing that LC symptoms were a sign of more serious pathologies, such as pulmonary embolism or myocarditis.^30^^,^^44^ HCWs sought advice and reassurance from colleagues with similar symptoms or who were working with people with LC.^30^^,^^44^ These connections were a key source of information given the lack of official government guidance or care pathways at the time.^30^^,^^44^ They felt let down by their national healthcare system because of the lack of support when trying to access care.^30^^,^^44^ Many also feared or experienced disapproval when trying to take time off work from their colleagues who did not believe in their symptoms.^30^^,^^44^ When reflecting on what services should be available for people suffering with LC, HCWs in both studies advocated for a ‘one-stop-shop’, multi-disciplinary team approach, which would help to fix the fragmented service they experienced and would offer personalized support for symptoms.^30^^,^^44^

A mixed methods study conducted in the UK between June 2021 and July 2022 also presented insights into HCWs’ lived experiences with LC and returning to work.^77^ Approximately two-thirds of their sample had taken sick leave due to LC (216/457), with 18% reporting that they were unable to return to work.^77^ The most important factors in returning to work were the nature and severity of LC symptoms, the availability of workplace supports, the specific demands and expectations of the role and HCWs own ability to manage their symptoms.^77^ Many said that others often attributed their LC symptoms to mental rather than physical causes, and around a third reported feeling unsupported at work when trying to return.^77^ Finally, staff reported mixed experiences with occupational health services, with some reporting positive experiences, and others saying that the services offered little in terms of advice, referrals and workplace adjustments.^77^

## Discussion

Our review highlighted a key issue in how LC is defined in the literature. The terms used in the studies included LC, OSC and PCS from NICE,^1^ post-COVID-19 condition from the WHO^3^, and post-acute COVID-19 and chronic COVID from Greenhalgh et al.^79^ Other terms common in the literature include but are not limited to long-haul COVID, long-term effects of COVID, and post-acute sequelae of SARS CoV-2 infection (PASC).^9^^,^^80^ Additionally, each of the studies defined LC using a different set of symptoms, with a minority failing to report what symptoms they measured. In total, 76 symptoms were associated with LC and none of these symptoms were reported in all of the 41 studies. This variety in the definitions and symptoms of LC is not unique to research on HCWs, and constitutes a significant challenge to comparing and consolidating results across LC studies.^4^ This is further shown in the wide variation in the prevalence of OSC and PCS reported by the studies, which were also likely confounded by poor sampling strategies. Given this, we recommend that future research in this area should converge towards a standard definition and measurement of LC. We discuss this recommendation further this later in this article.

As for risk factors, it is difficult to speculate why certain characteristics are associated with increased odds of developing LC without a known mechanism or pathogenesis. Other papers consider this in more detail in the context of proposed mechanisms for the condition.^14^^,^^81^^,^^82^ Comparing the results of the included studies with a meta-analysis that explored risk factors for LC among the general population in 41 studies,^14^ we note that many significant risk factors are also present in the current review. Additionally, the direction of statistical significance in also consistent with the results of the meta-analysis, e.g. some of studies found that females and those of older age were at increased risk of developing LC in both reviews. We note that certain characteristics may require further consideration among HCWs, particularly by means of a meta-analysis, which would help to ascertain whether these characteristics may be true risk factors across studies.^21^ We also note that the variable definitions of LC in the included studies would make it complicated to conduct a meta-analysis of the data due to heterogeneity. Additionally, emerging research indicates that common mental disorders may be risk factors for LC.^14^ Despite this, only two studies in the current review explored this, with one finding a history of depression to be a significant risk factor for developing LC^35^ and the other finding that anxiety was a significant risk factor.^73^

Research on the impacts of LC among HCWs was lacking. Among the quantitative studies, the longitudinal research offered the most robust findings, though these only accounted for a small number of included studies. They highlighted negative impacts such as reduced general health,^28^ and quality of life,^28^^,^^29^^,^^71^ and increased functional impairment^71^ and challenges in returning to work.^33–35^ In the UK, the Industrial Injuries Advisory Council have reported on research exploring the impacts of LC within the general public, highlighting findings from the ONS that 21% of people with LC reported that prolonged symptoms had disrupted their daily activities ‘a lot’.^15^ They also report on findings that conditions such as pulmonary fibrosis, pneumonitis, pulmonary embolism, ischemic stroke, myocardial infarction and post-intensive care syndrome are also prominent among people with LC,^15^ which were largely absent in the current review. Much of the cross-sectional research in this review failed to explore the impacts that prolonged symptoms had on HCWs’ personal and professional lives. Qualitative evidence was confined to three studies, though all three offered important insights into HCWs’ lived experience of LC in the UK. Further qualitative work on patient groups with LC has uncovered additional challenges such as limits to daily functioning, psychological impacts of prolonged symptoms, and learning to cope with new symptoms.^83–85^

### Strengths and limitations

A strength of the current review is the inclusion of 56 studies with a total of 60 810 HCWs working in 45 named countries. This allowed us to examine the broad range of symptoms and risk factors that have been explored in the literature internationally and may be associated with LC among HCWs. Additionally, while our review focused on research among HCWs, it is likely that the review offers insights to other occupational groups. This may be especially true for groups who were at higher risk to COVID-19 infection compared to other. Examples of these groups in the UK include educators, bus and coach drivers, and police and protective services staff.^15^

There were also limitations to our review. The principal limitation of our review was the lack of a standard definition for LC. As the included studies used a variety of definitions and symptoms to designate LC, the studies effectively measured different entities while all claiming to measure LC. This created significant challenges in comparing the prevalence, symptoms, and risk factors associated with LC. While we did not conduct a meta-analysis on the data, as our aim was to present a narrative overview of the research to date, we note that the heterogeneity observed in the included studies means that a meta-analysis would have been inappropriate. Regardless, the lack of a meta-analysis means we are unable to make claims about the prevalence, symptoms, or risk factors associated with LC in these studies beyond the basic overview presented in the results. Further reviews should devise stricter inclusion criteria for studies based on how they define LC, rather than including studies that purported to examine LC, as we had.

Another limitation is the lack of a formal quality assessment on the included studies. This was beyond the scope of the current review, though may have illuminated further insights, particularly if the evidence was found to be of particularly low quality. Additionally, due to time constraints, it was not within the scope of this review to conduct a cross-cultural examination to explore how geographical contexts may influence HCWs’ experiences with LC, e.g. access to occupational health services, specific LC health services or special provisions for LC-related sickness absence. Finally, our review highlights a number of gaps in the current literature, highlighting that there is much left to learn about LC. In the next section, we recommend how future research may address these gaps to further inform our understanding of LC among HCWs.

### Recommendations

Based on the evidence in our review and the gaps in the literature, we have four recommendations. As previously discussed, our first recommendation is that future research in this area should converge towards a standard definition and measurement of LC. One solution may be to adopt the core outcome set and Delphi study conducted for post-COVID-19 condition.^86^ Eleven outcomes achieved consensus for inclusion in this core outcome set, including fatigue; pain; post-exertion symptoms; work or occupational and study changes; survival; and functioning, symptoms, and conditions for each of cardiovascular, respiratory, nervous system, cognitive, mental health and physical outcomes.^86^ This would strengthen the findings of individual studies, as well as allow for easier comparisons between studies. We also advise that future reviews, particularly those including a meta-analysis, adopt more specific inclusion criteria when defining LC and identifying research articles.

Secondly, further longitudinal research is required, with greater focus on exploring the impacts of LC among HCWs and the prognosis of people experiencing LC. As HCWs are an occupational group, more focus should also be paid to exploring the impact of LC on their work participation. This should include research on absenteeism and presentism, as well as redeployment and medical retirement attributable to LC. This may lead to the discovery of patterns of people whose ability to work may be most impacted by LC and may be useful in allocating support to help those most affected by COVID-19 to remain productive in the workforce.

Thirdly, more qualitative work is required, as it would allow for an in-depth exploration of the impact of prolonged symptoms on HCWs’ personal and professional lives. The two qualitative studies lacked perspectives on the lived experience of returning to healthcare work following LC,^30^^,^^44^ as well as information on managing symptoms and symptom recovery and relapses. Additionally, both studies were based in the UK and are likely outdated. For example, HCWs called for LC-dedicated healthcare services following their experiences with care in 2020,^30^^,^^44^ which are now accessible in the UK^87^. A mixed methods study from the UK offered insights into return to work,^77^ though evidence from other countries is needed. More qualitative work may likely also highlight potential differences between professions within healthcare, a finding which is currently largely absent, as well as allow for a more nuanced cross-cultural investigation of how access to certain supports, such as LC clinics or Occupational Health departments, may impact HCWs.

Finally, the review did not include any research examining treatments or access to occupational health supports for HCWs with LC. These supports are important to ensure that HCWs, as well as people working in other industries, are supported in managing their symptoms and remain in work. Studies designing and measuring the effectiveness of these supports in across occupational settings is timely.

## Conclusion

This review examined the definitions, prevalence, symptoms, risk factors and impacts associated with LC in the literature among HCWs. The cause of LC remains unclear and may remain so for some time. Until further research leads to a more specific pathogenesis and treatment, HCW with LC should have access to dedicated LC services and occupational health support, so as to receive expert advice on the management of their symptoms.

## Supplementary Material

BMB_LC_in_HCWs_Review_v2_ldae008_0_Appendices_ldae008

## Data Availability

The data underlying this article are available in the article and in its online supplementary material.
