# Supplementary material for: Long COVID among healthcare workers: a narrative review of definitions, prevalence, symptoms, risk factors and impacts
Source: Br Med Bull. 2024 Aug 25;151(1):16–35. doi: 10.1093/bmb/ldae008 (PMC11436955; doi:10.1093/bmb/ldae008)
Supplement: BMB_LC_in_HCWs_Review_v2_ldae008_0_Appendices_ldae008 [file bmb_lc_in_hcws_review_v2_ldae008_0_appendices_ldae008.docx]

**Long COVID among Healthcare Workers: A narrative review of definitions, prevalence, symptoms, risk factors, and impacts.**

**Appendices**

**Appendix 1:** Completed Database Searches

| **Search in OVID (EMBASE, MedLine, PsycInfo)** | |
| --- | --- |
| **First search:** 5,502 results (1pm, January 9^th^, 2023) | **Second search:** 7,387 results (1:30pm, May 24^th^, 2024) |
| ("healthcare workers" or "health care workers" or "health workers" or "healthcare staff" or "health care staff" or "health staff" or "healthcare personnel" or "health care personnel" or "health personnel" or "healthcare professionals" or "health care professionals" or "health professionals" or "healthcare practitioner*" or "health care practitioner*" or "health practitioner*" or nurse* or doctor* or medic* or midwif* or midwives or physician* or clinician* or paramedic* or therapist* or "allied health professional*").af. and ("long COVID" or "long-COVID" or "long corona" or "long-corona" or "longcorona" or "post-COVID syndrome" or "post COVID syndrome" or "postCOVID syndrome" or "post-COVID-19 syndrome" or "post COVID-19 syndrome" or "postCOVID-19 syndrome" or "ongoing symptomatic covid*" or "post-COVID sequelae" or "post-acute sequelae of COVID" or "post-acute sequelae of corona" or "post-acute sequelae of Sars-Cov").af. and (experienc* or prevalence or impact* or symptom* or fatigue* or weakness or breathlessness or impaired or dysfunction or pain or anxiety or depress* or myalgia or cough or sweat* or dizz* or affected or vertigo or appetite or fever or nausea or vomit* or dyspnea or dyspepsia or "brain fog" or hospitalis* or hospitaliz* or recover* or predictor* or "co-morbidit*").af. | |
| **Search in CINAHL (via EBCSO) – 398 results – 4pm, 9^th^ of January, 2023** | |
| **First search:** 398 results (4pm, January 9^th^, 2023) | **Second search:** 409 results (2pm, May 24^th^, 2024) |
| TX ( "healthcare workers" or "health care workers" or "health workers" or "healthcare staff" or "health care staff" or "health staff" or "healthcare personnel" or "health care personnel" or "health personnel" or "healthcare professionals" or "health care professionals" or "health professionals" or "healthcare practitioner*" or "health care practitioner*" or "health practitioner*" or nurse* or doctor* or medic* or midwif* or midwives or physician* or clinician* or paramedic* or therapist* or "allied health professional*" ) AND TX ( "long COVID" or "long-COVID" or "long corona" or "long-corona" or "longcorona" or "post-COVID syndrome" or "post COVID syndrome" or "postCOVID syndrome" or "post-COVID-19 syndrome" or "post COVID-19 syndrome" or "postCOVID-19 syndrome" or "ongoing symptomatic covid*" or "post-COVID sequelae" or "post-acute sequelae of COVID" or "post-acute sequelae of corona" or "post-acute sequelae of Sars-Cov" ) AND TX ( experienc* or prevalence or impact* or symptom* or fatigue* or weakness or breathlessness or impaired or dysfunction or pain or anxiety or depress* or myalgia or cough or sweat* or dizz* or affected or vertigo or appetite or fever or nausea or vomit* or dyspnea or dyspepsia or "brain fog" or hospitalis* or hospitaliz* or recover* or predictor* or "co-morbidit*" ) | |
| **Search PubMed (via NCBI) – 2,355 results – 4:20pm, 9^th^ of January, 2023** | |
| **First search:** 2,355 results (4:20pm, January 9^th^, 2023) | **Second Search:** 2,727 results (2:20pm, May 24^th^, 2024) |
| (("healthcare workers" or "health care workers" or "health workers" or "healthcare staff" or "health care staff" or "health staff" or "healthcare personnel" or "health care personnel" or "health personnel" or "healthcare professionals" or "health care professionals" or "health professionals" or "healthcare practitioner*" or "health care practitioner*" or "health practitioner*" or nurse* or doctor* or medic* or midwif* or midwives or physician* or clinician* or paramedic* or therapist* or "allied health professional*") AND ("long COVID" or "long-COVID" or "long corona" or "long-corona" or "longcorona" or "post-COVID syndrome" or "post COVID syndrome" or "postCOVID syndrome" or "post-COVID-19 syndrome" or "post COVID-19 syndrome" or "postCOVID-19 syndrome" or "ongoing symptomatic covid*" or "post-COVID sequelae" or "post-acute sequelae of COVID" or "post-acute sequelae of corona" or "post-acute sequelae of Sars-Cov")) AND (experienc* or prevalence or impact* or symptom* or fatigue* or weakness or breathlessness or impaired or dysfunction or pain or anxiety or depress* or myalgia or cough or sweat* or dizz* or affected or vertigo or appetite or fever or nausea or vomit* or dyspnea or dyspepsia or "brain fog" or hospitalis* or hospitaliz* or recover* or predictor* or "co-morbidit*") | |

**Appendix 2:** Inclusion and exclusion criteria for the review.

| **Inclusion Criteria:** | **Exclusion Criteria:** |
| --- | --- |
| **Population:** | **Population:** |
| - Studies with HCWs, either clinical or non-clinical, who developed long COVID. - Human subjects. | - Studies with HCWs who did not develop long COVID. - Studies with people who developed long COVID but were not HCWs. - Studies with people who developed long COVID, including HCWs, but do not present separate results for this group. |
| **Phenomenon of Interest:** | **Phenomenon of Interest:** |
| - The symptoms and prevalence of long COVID among HCWs. - The impact of long COVID on the lives of HCWs. - The impact of long COVID among HCWs on healthcare systems. - Characteristics that are associated with the development of ongoing symptomatic COVID-19, between 4 and 12 weeks after initial infection. - Characteristics that are associated with the development of post-COVID syndrome, 12 weeks or more after initial infection. | - Studies that do not explore any of the listed aspects of interest about long COVID. - Studies that look only at the experience of working in healthcare during the COVID-19 pandemic. |
| **Study Design:** | **Study Design:** |
| - Cross-sectional or longitudinal studies that explore HCWs’ experiences with long COVID. - Any other design which explores the nature, prevalence, or impacts of long COVID among a sample of HCWs. - Qualitative or mixed methods studies that explore HCWs’ experiences with long COVID. - Papers that are published as peer-reviewed articles or have not yet been published but have been shared as a pre-print. | - Case reports or case series. - Reviews of any type (e.g., literature review, systematic review, etc.). - Methodological or theoretical papers about long COVID. - Papers that seek to predictively model long COVID instead of using empirical data. - Conference proceedings and abstracts, editorials, commentaries, and letters. |

**Appendix 3:** Draft table showing information to be extracted from the included studies.

| **Charting Elements** | **Study Characteristics** |
| --- | --- |
| Reference details | Study ID (assigned by authors) |
|  | Author(s) |
|  | Study title |
|  | Journal (volume and issue) |
|  | Year of publication |
|  | DOI |
| Study Context | Study aim(s)/objective(s) |
|  | Country |
|  | Sample characteristics (age, gender, ethnicity, sample size, existing co-morbidities, vaccination status) |
|  | Professions |
|  | Workplace setting |
|  | Timeframe of data collection |
| Study Design | Methodology |
|  | Method of recruitment |
|  | Method of data collection |
|  | Method of analysis |
| Results | Definition of long COVID |
|  | Prevalence of developing long COVID |
|  | Symptoms associated with long COVID and their prevalence |
|  | Characteristics associated with ongoing symptomatic COVID |
|  | Characteristics associated with post COVID-19 condition |
|  | Characteristics associated with long COVID (stage not specified) |
|  | Impacts of long COVID among HCWs on their own personal lives |
|  | Impacts of long COVID among HCWs on their own professional lives |
|  | Impacts of long COVID among HCWs on the healthcare system |
